# Partitioning risk factors for embolic stroke of undetermined source using exploratory factor analysis

**DOI:** 10.1177/17474930211009847

**Published:** 2021-04-26

**Authors:** Jon D Perkins, Naveed Akhtar, Rajvir Singh, Asad Kamran, Saadat Ilyas

**Affiliations:** 1 Neuroscience Institute, Hamad General Hospital, Doha, Qatar; 2 PMARC, University of Edinburgh, Edinburgh, UK; 3 Weill Cornell Medicine, Doha, Qatar; 4 Heart Hospital, 36977 Hamad Medical Corporation, Doha, Qatar

**Keywords:** Stroke, ESUS, exploratory factor analysis, cardiac dysfunction, risk model

## Abstract

**Background:**

Embolic stroke of undetermined source (ESUS) accounts for up to 25% of strokes. Understanding risk factors associated with ESUS is important in reducing stroke burden worldwide. However, ESUS patients are younger and present with fewer traditional risk factors. Significant global variation in ESUS populations also exists making the clinical picture of this type of stroke unclear.

**Methods and results:**

ESUS patients were pair matched for age, sex, and ethnicity with a group of all other strokes (both *n* = 331). Exploratory factor analysis was applied in both groups to 14 risk and clinical factors to identify latent factors. In ESUS patients, two latent factors emerged consisting primarily of heart-related variables such as left ventricular wall motion abnormalities, reduced ejection fraction, and increased left atrial volume index, as well as aortic arch atherosclerosis. This is in comparison to the all other strokes group, which was dominated by traditional stroke risk factors.

**Conclusions:**

Our findings support the existence of a unique pattern of risk factors specific to ESUS. We show that LVWMA and corresponding changes in left heart function are a potential source of emboli in these patients. In addition, the clustering of aortic arch atherosclerosis with left heart factors suggests a causal link. Through the application of exploratory factor analysis, this work contributes to a further understanding of stroke mechanisms in ESUS.

## Introduction

Since its formal characterization,^1^ embolic stroke of undetermined source (ESUS) has generated much interest. Several risk factors have been associated with an ESUS diagnosis, such as aortic arch atherosclerosis,^2^ hypertension,^3^ and left ventricular wall motion abnormalities (LVWMA).^4^ However, the clinical picture of ESUS remains unclear with covert atrial fibrillation the most widely speculated upon cause.^5,6^

One of the difficulties in identifying risk profiles for ESUS patients is the consistent finding that they are younger and carry fewer traditional risk factors than other forms of stroke diagnosis.^7^ Another difficulty is the global variation in ESUS populations, likely stemming from genetic and culturally determined factors.^8,9^ In addition, the majority of work on this topic uses multivariate statistics to show how risk factors differ from other forms of stroke diagnoses (or normal populations) but not how they manifest in ESUS only populations.

To address these issues, this study applies exploratory factor analysis to investigate the interactions between risk factors for ESUS while controlling for age, sex, and ethnicity. Exploratory factor analysis* assesses underlying interactions between variables and specifies latent factors.*^10^ Latent (meaning “hidden”) variables are unobserved but identified through shared features of observed variables. The usefulness of understanding risk factor interactions in this way is highlighted by metabolic syndrome, whereby vascular risk and metabolic abnormalities combine to confer increased cerebro- and cardiovascular hazard.^11^ To our knowledge, no studies have explored similar clustering of risk factors in ESUS patients and this work is the first to do so.

## Methods

Stroke data were prospectively collected at a tertiary referral center between 1 January 2017 and 31 March 2020. Stroke subtypes were classified according to TOAST criteria by stroke neurologists.^12^ ESUS diagnosis was confirmed using criteria described by Hart et al.^1^ Each patient underwent vascular imaging using cervico–cranial magnetic resonance angiography, computed tomography angiography, digital subtraction angiography, and/or carotid Doppler studies. Transthoracic echocardiogram and Holter for at least 24–48 h were also performed. Patients with large PFO were excluded.

Seventeen risk factors were studied as determined by the literature and stroke neurologists. These were; age, sex, and ethnicity (used for pair matching), hypertension (HTN), diabetes mellitus (DM), dyslipidemia (DYSLIP), smoking, coronary artery disease (CAD), left atrial volume index (LAVI), ejection fraction (EF), left ventricular diastolic dysfunction (LVDD), mitral calcification (MITCAL), aortic arch atherosclerosis (AAA), left ventricular wall motion abnormalities (LVWMA), aortic root diameter (ART), left ventricular mass index (LVMI), and regional wall thickness (RWT).

### Data analysis

Where appropriate, variables were converted to binary or categorical outcomes. Two data sets were created; one composed of ESUS patients and the second, all other strokes. All other strokes consisted of non-cardioembolic, multiple etiologies, and cardioembolic strokes. All other strokes served as a comparator to validate the underlying pattern of clinical and risk features identified in ESUS were unique to that diagnosis. Pair matching was based first on sex, then country of origin, followed by age (within two years). Unless a match for all three was found, the patient was excluded.

### Statistical analysis

Descriptive analysis was performed using Statistical Package for Social Sciences Version 24.0.^13^ Continuous data are presented as mean and standard deviation (M, ±SD) with absolute numbers and percentage reported for categorical variables. To explore differences in clinical and risk factors between ESUS and all other strokes, N-1 Chi square test of proportions was used.^14^

Exploratory factor analysis was applied to each data set using M*plus* version 6.12, with mean- and variance-corrected-weighted least squares (WLSMV) and Geomin rotation.^15^ Model fit cut-off values were ≥.90 for comparative fit index (CFI) and Tucker Lewis index (TFI) with root mean square error of approximation (RMSEA) ≤.08. Loadings ≥.3 indicated satisfactory contribution to a latent variable.^10^ See Supplementary Material 1 for full description of exploratory factor analysis.

## Results

From 2706 stroke patients, 654 (24.2%) were diagnosed with ESUS. Excluded were 196 (30.0%) for missing data and 127 (27.7%) where no match could be found leaving 331 ESUS patients coupled with 331 all other strokes. In all other strokes, 244 (73.7%) were non-cardioembolic (large artery disease and small vessel disease), 50 (15.1%) multiple aetiologies, and 37 (11.2%) cardioembolic. Mean age was the same for each group (51.1 years) with approximately half of patients above 55 years (48.3% vs. 51.7%). Males were 89.4% in both groups with Indians the largest nationality 104 (31.4%). More ESUS patients had LVWMA (*p* = .001), EF below 30.0 (*p* = .007), LAVI above ≥40 mL/m^2^ (*p* = .005), and severe LVDD (*p* = .024). In contrast, all other stroke patients had more severe RWT (*p* = .001) (Table 1).

**Table 1.** Demographic, clinical, and radiological/echocardiographic characteristics of ESUS and all other stroke groups.

		ESUS		AOS		
Variable		Total *N*	Mean/No. (SD/%)	Total *N*	Mean/No. (SD/%)	*p*-value
Demographic						
Age	All	331	55.1 ( ± 12.2)	331	55.1 ( ± 12.1)	0.99
	>55		160 (48.3)		163 (49.2)	0.82
	≤55		171 (51.7)		168 (50.8)	0.82
Sex	Male	331	281 (84.9)	331	281 (84.9)	–
	Females		50 (15.1)		50 (15.1)	–
Country	India	331	104 (31.4)	331	104 (31.4)	–
	Qatar		62 (18.7)		62 (18.7)	–
	Pakistan		29 (8.8)		29 (8.8)	–
	Bangladesh		29 (8.8)		29 (8.8)	–
	Other^a^		107 (32.3)		107 (32.3)	–
Clinical						
Stroke	NCE	N/A	–	331	244 (73.7)	–
	CE		–		37 (11.2)	–
	Multiple aetiologies		–		50 (15.1)	–
HTN		331	269 (81.3)	331	260 (78.5)	0.38
Diabetes		331	196 (59.2)	331	204 (61.6)	0.52
Smoking		322	51 (15.8)	327	37 (11.3)	0.09
CAD		331	106 (32.0)	331	110 (33.2)	0.74
DYSLIP		330	237 (71.8)	331	254 (76.7)	0.15
Radiological/echocardiographic						
LAVI	Normal	331	230 (69.5)	331	232 (70.1)	0.87
	Mild		34 (10.3)		48 (14.5)	0.10
	Moderate		32 (9.7)		30 (9.1)	0.79
	Severe		35 (10.6)		21 (6.3)	0.05**
LVWMA	None	331	208 (62.8)	331	282 (85.2)	0.001*
	Focal		93 (28.1)		31 (9.4)	0.001*
	Global		30 (9.1)		18 (5.4)	0.07
EF	≤29.9	331	16 (4.8)	331	4 (1.2)	0.001*
	30–40		37 (11.2)		24 (7.3)	0.16
	41–51		100 (30.2)		80 (24.2)	0.08
	≥52		178 (53.8)		223 (67.4)	0.001*
LVDD	Normal	330	108 (32.6)	330	120 (36.4)	0.32
	Mild		154 (32.7)		165 (50.0)	0.40
	Moderate		53 (16.1)		40 (12.1)	0.14
	Severe		15 (4.5)		5 (1.5)	0.02**
RWT	Normal	331	110 (33.2)	331	41 (12.4)	0.001*
	Abnormal		177 (53.5)		183 (55.3)	
	Severe		44 (13.3)		107 (32.3)	
LVMI	Normal	331	318 (96.1)	331	320 (96.7)	0.67
	Abnormal		13 (3.9)		11 (3.3)	0.67
MITCAL		331	74 (22.4)	330	67 (20.3)	0.51
ARD		323	3.1 (±0.52)	325	3.1 (±0.51)	0.77
AAA		331	117 (35.3)	331	106 (32.0)	0.37

AOS: all other strokes; NCE: non-cardioembolic stroke; CE: cardioembolic; DYSLIP: dyslipidemia; LVWMA: left ventricular wall motion abnormalities; MITCAL: mitral calcification; CAD: coronary artery disease; RWT: regional wall thickness; ARD: aortic root diameter; AAA: aortic arch atherosclerosis.

a Others include; Philippines, Egypt, Sri Lanka, Sudan, Syria, Oman, Yemen and Nepal. **p<0.05, *p<0.001.

**Figure 1.**
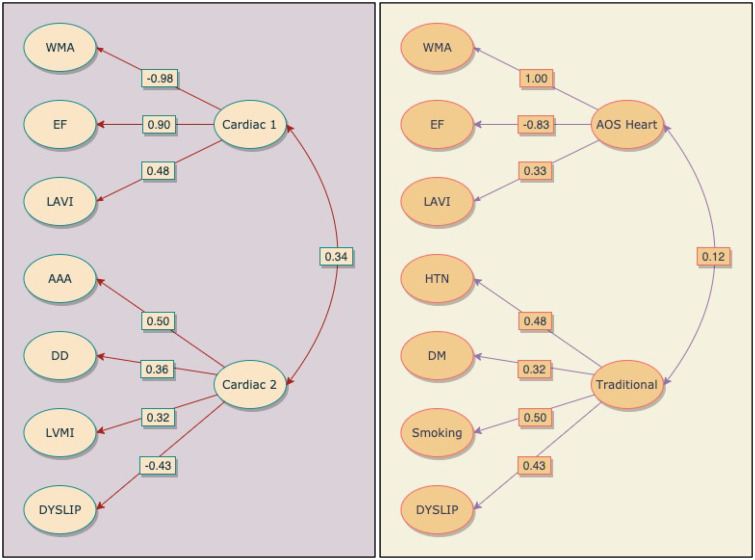
Schematic representation of latent variables in the final ESUS (left) and all other strokes (right) models. Indicator variables appear on the left of each diagram and latent variables on the right. Arrows point to indicators that cluster with latent variables and each loading is displayed in the boxes. Between latent variables, double-headed arrows indicate correlation with r-values shown in the box. AAA: aortic arch atherosclerosis; DYSLIP: dyslipidemia; WMA: wall motion abnormalities; AOS: all other strokes.

In the ESUS group, a final two-factor model was identified (χ^2 ^= 56.4, df, 53, RMSEA, 0.01, CFI, 1.00, TFI, 1.00). In the first latent factor “Cardiac 1”, the highest loading variable was LVWMA (− 0.98), which was negatively correlated (LVWMA were coded 0 –2 for none, focal, and global) followed by EF (0.71) and LAVI (0.48) (see Figure 1, Table 2). The second latent factor “Cardiac 2” also included heart-related features, LVDD (0.36), and LVMI (0.32) clustered with aortic arch atherosclerosis (0.50), and dyslipidemia (−0.43). Aortic arch atherosclerosis was the dominant Cardiac 2 contributor and the only positively loading non-heart feature found in ESUS patients. The correlation between Cardiac 1 and Cardiac 2 was moderate (0.34).

**Table 2.** Geomin rotated factor loadings for final ESUS and all other strokes models.

	ESUS		AOS	
Variable	Latent factor 1	Latent factor 2	Latent factor 1	Latent factor 2
LVWMA	**−0.98**	−0.01	**1.00**	−0.01
EF	**0.90**	−0.07	**−0.83**	0.12
LAVI	**0.48**	−0.03	**0.33**	−0.02
HTN	0.27	0.05	0.01	**0.48**
DM	0.27	−0.01	−0.05	**0.32**
SMOKING^a^	0.36	0.39	0.27	**0.50**
DYSLIP	0.01	**−0.43**	−0.29	**0.43**
AAA	−0.08	**0.50**	0.06	−0.29
LVDD	0.19	**0.36**	0.24	0.07
LVMI	0.19	**0.32**	0.16	0.21
MITCAL^b^	0.26	0.22	0.30	−0.41
CAD	0.06	−0.04	0.07	0.10
RWT	0.14	−0.14	0.01	0.05
ARD	−0.03	0.22	0.04	0.01

AOS: all other strokes; MITCAL: mitral calcification; AAA: aortic arch atherosclerosis; RWT: regional wall thickness; ARD: aortic root diameter; DYSLIP: dyslipidemia; LVWMA: left ventricular wall motion abnormalities.

Note: Loadings ≥.3 are in bold.

a Deleted in second ESUS analysis.

b Deleted in second analysis of all other strokes.

In the all other strokes data, a two-factor model also emerged (χ^2 ^= 64.7, df, 53, RMSEA, 0.03, CFI, 0.98, TFI, 0.97). The first latent factor “AOS Heart” mirrored ESUS findings and included LVWMA (1.0) and EF (−0.83) but loaded in reverse meaning that normal or focal/ LVWMA and normal or mild/EF, clustered with the third contributor, LAVI (0.48), (see Figure 1, Table 2). A second latent factor “Traditional” consisted entirely of tradition risk factors. The highest loading variable was smoking (0.50), followed by HTN (0.48), dyslipidemia (0.43), and DM (0.32). The correlation between AOS Heart and Traditional latent factors was small at 0.12. A full description of results can be found in Supplementary Material 1.

## Discussion

The aims of this work were to explore the underlying relationships between different risk factors for ESUS. After the exploratory factor analysis, two latent factors emerged in both ESUS and all other strokes groups with unique patterns of risk observed despite being matched for age, sex, and ethnicity. As the pattern unique to ESUS was predominantly heart related, it indicates a cardioembolic source of emboli in these patients.

As mentioned, ESUS patients are younger and consequently have fewer traditional risk factors.^7,9^ In this work, 48.3% of participants were over 55 years of age and yet traditional risk factors were absent in the ESUS latent factors (dyslipidemia loaded negatively in Cardiac 2 suggesting abnormal blood lipids are not involved in ESUS pathology). This finding cannot be attributed to differences in sex or ethnicity as these were controlled for and contrasts starkly with the all other strokes group where traditional risk factors clustered as an independent latent factor. This is significant as it is evidence of a pattern of risk features that occur in ESUS patients of any age. This pattern of risk may extend to ethnicity, which was similarly controlled. A question of interest is whether Caucasians would produce comparable findings? Data presented here suggest this may be so as wide ethnic disparities exist between the South East and Western Asians in this sample. In addition, the risk factors found in this work have been reported in Caucasians elsewhere and further studies are warrented.^2,8^

Of the two latent factors identified in ESUS patients, Cardiac 1 was the largest contributor and included the solely heart-related factors, LVWMA, LAVI, and EF. LVWMA are both associated and predictive of EF and increased LAVI is reported in patients with wall motion problems.^16-18^ ESUS Cardiac 1 differed from all other strokes where LVWMA were absent or minimally involved in stroke as was reduced EF. There is growing evidence of a role for LVWMA in ESUS pathology.^4,19,20^

The finding of predominantly heart-related clustering extended to Cardiac 2 where reduced LVDD and increased LVMI featured. The moderate correlation between Cardiac 1 and 2 reflects an association between these variables and Cardiac 1 items. For instance, LVDD and LVWMA are associated with ventricular remodelling after myocardial infarction and increased atrial volume occurs in response to the additional burden in regulating ventricular filling LVDD causes.^21,22^ LVWMA have also been linked to diastolic dysfunction and ventricular enlargement independently.^4,9,23^ A final risk factor in Cardiac 2 was aortic arch atherosclerosis, which has previously been identified in ESUS patients and is discussed further below.^2,24^

An advantage of exploratory factor analysis is that after isolating risk factors we may speculate on how variables influence each other and importantly, on the sequence of interactions that may cause pathology. In the ESUS group, the highest loading variable, and the one that underpins other conspicuous risk and clinical factors, was LVWMA. LVWMA are sensitive indicators of myocardial ischemia suggesting that many of these patients had previous or unrecognized myocardial infarction.^25^

An interesting question from Cardiac 2 is why aortic arch atherosclerosis and LVDD were coupled? We speculate that it relates to altered hemodynamics from poor left heart function with increased likelihood of blood stagnation and disturbed flow.^26,27^ Changes in hemodynamics are a well-described cause of atherosclerotic plaque through pathways such as; increased exposure of atherosclerotic initiators with the vessel wall, changes in wall shear stress and activation of flow-sensitive coding and non-coding genes.^28–30^ Although flow was not directly measured in this work, it is a plausible explanation for the aortic arch atherosclerosis-LVDD clustering presented here and future studies of flow dynamics in ESUS patients are of interest. Furthermore, the location of plaque formation is not random, including in the aorta.^31,32^ This implies that a distinct pattern of aortic atheroma may be present if altered flow dynamics are a cause of plaque in ESUS. In our model, approximately one-third of ESUS patients had aortic arch atherosclerosis suggesting a role as a source of emboli. However, the detection and categorization of aortic arch atherosclerosis require transesophageal echocardiography (TEE), which is limited in ESUS studies as shown by the NAVIGATE ESUS trial where only 19% of patients underwent TEE.^2,33^ Our work also highlights the importance of TEE in ESUS.

Left heart dysfunction and resultant hemodynamic changes are also associated with hypercoagulability and increased likelihood of thrombus formation.^34,35^ Ventricular thrombi are common after myocardial infarction and almost always appear at the site of LVWMA.^36^ However, thrombi are sometimes missed in patients with ESUS for example, Takasugi et al. report a LV thrombus detection rate of 20-1 using cardiac magnetic resonance imaging versus TEE.^37^

LV dysfunction influences atrial hemodynamics and function as indicated in our model by the clustering of LAVI with LVWMA and other measures of left heart dysfunction.^38,39^ Atrial thrombi are a source of embolic stroke and a topic of great interest to ESUS research due to the often-seen atrial dysfunction (atrial fibrosis, left atrial size, and increased LAVI) observed in these patients.^40,41^ Currently, patients with atrial cardiomyopathy are being investigated for anticoagulants versus aspirin (the ARCADIA trial) and left atrial appendage flow velocity ≤0.2 m/sg (the ATTICUS trial) which will shed further light on the role of the left atrium in ESUS patients.^42,43^ An intriguing outcome regarding the NAVIGATE and RE-SPECT ESUS trials was that both failed to find a difference in anticoagulants versus antiplatelets in ESUS, which could be explained by some patients presenting with either atherosclerotic aetiologies versus thrombosis.^33,44^ Support for this proposition comes from the COMPASS trial where a combined low dose of rivaroxaban and aspirin was found to reduce stroke compared to either medication as monopharmacy.^45^

## Limitations

This study has several limitations. It is a retrospective analysis of prospectively collected data, therefore collection and registration bias may be present. As this study used a paired match design, the number of excluded participants was high. Only recorded indicator variables are included, and unregistered significant variables may have been omitted. Future work may need to explore other variables as ESUS data accumulates. Females were less than males due to expatriate population in Qatar. Despite these limitations, our study is the first to our knowledge to use exploratory factor analysis to investigate risk and clinical factors in patients with ESUS and provides the only model of how these factors interact in this group.

## Supplemental Material

sj-pdf-1-wso-10.1177_17474930211009847 - Supplemental material for Partitioning risk factors for embolic stroke of undetermined source using exploratory factor analysis Click here for additional data file. Supplemental material, sj-pdf-1-wso-10.1177_17474930211009847 for Partitioning risk factors for embolic stroke of undetermined source using exploratory factor analysis by Jon D Perkins, Naveed Akhtar, Rajvir Singh, Asad Kamran and Saadat Ilyas in International Journal of Stroke

## References

[1] Hart RG Diener H-C Coutts SB , et al. Embolic strokes of undetermined source: the case for a new clinical construct. Lancet Neurol 2014; 13: 429–438. 24646875 10.1016/S1474-4422(13)70310-7

[2] Ntaios G Pearce LA Meseguer E , et al. Aortic arch atherosclerosis in patients with embolic stroke of undetermined source: an exploratory analysis of the NAVIGATE ESUS trial. Stroke 2019; 50: 3184–3190. 31526123 10.1161/STROKEAHA.119.025813

[3] Ntaios G Perlepe K Lambrou D , et al. Prevalence and overlap of potential embolic sources in patients with embolic stroke of undetermined source. JAHA 2019; 8: e012858. 31364451 10.1161/JAHA.119.012858 PMC6761628

[4] Kamran S Akhtar N George P , et al. Embolic pattern of stroke associated with cardiac wall motion abnormalities; narrowing the embolic stroke of undetermined source category. J Stroke Cerebrovasc Dis 2020; 29: 104509. 31759913 10.1016/j.jstrokecerebrovasdis.2019.104509

[5] Sanna T Diener H-C Passman RS , et al. Cryptogenic stroke and underlying atrial fibrillation. N Engl J Med 2014; 370: 2478–2486. 24963567 10.1056/NEJMoa1313600

[6] Verma N Ziegler PD Liu S Passman RS . Incidence of atrial fibrillation among patients with an embolic stroke of undetermined source: insights from insertable cardiac monitors. Int J Stroke 2019; 14: 146–153. 30196791 10.1177/1747493018798554

[7] Hart RG Catanese L Perera KS , et al. Embolic stroke of undetermined source. Stroke 2017; 48: 867–872. 28265016 10.1161/STROKEAHA.116.016414

[8] Kasner SE Lavados P Sharma M , et al. Characterization of patients with embolic strokes of undetermined source in the NAVIGATE ESUS randomized trial. J Stroke Cerebrovasc Dis 2018; 27: 1673–1682. 29525076 10.1016/j.jstrokecerebrovasdis.2018.01.027 PMC6701183

[9] Perkins JD Akhtar N George P , et al. Prevalence, characteristics and risk factors for embolic stroke of undetermined source in West and South Asia and North African population residing in Qatar. J Stroke Cerebrovasc Dis 2020; 29: 104666. 32165099 10.1016/j.jstrokecerebrovasdis.2020.104666

[10] Tabachnick BG Fidell LS . Using multivariate statistics, 5th ed. Pearson: Needham Heights, MA, 2001.

[11] Arenillas JF Moro MA Dávalos A . The metabolic syndrome and stroke. Stroke 2007; 38: 2196–2203. 17540972 10.1161/STROKEAHA.106.480004

[12] Adams HP Bendixen BH Kappelle LJ , et al. Classification of subtype of acute ischemic stroke. Definitions for use in a multicenter clinical trial. TOAST. Trial of Org 10172 in Acute Stroke Treatment. Stroke 1993; 24: 35–41. 7678184 10.1161/01.str.24.1.35

[13] IBM Corp . Released 2016. *IBM SPSS statistics for windows, version 24.0*, Armonk, NY: IBM Corp, 2016.

[14] Campbell I . Chi-squared and Fisher-Irwin tests of two-by-two tables with small sample recommendations. Stat Med 2007; 26: 3661–3675. 17315184 10.1002/sim.2832

[15] Muthén LK Muthén BO . Mplus user's guide, 6th ed. Los Angeles, CA: Muthén & Muthén, 2011.

[16] Nowosielski M Schocke M Mayr A , et al. Comparison of wall thickening and ejection fraction by cardiovascular magnetic resonance and echocardiography in acute myocardial infarction. J Cardiovasc Magn Reson 2009; 11: 22. 19589148 10.1186/1532-429X-11-22 PMC2717065

[17] Lebeau R Serri K Morice MC , et al. Assessment of left ventricular ejection fraction using the wall motion score index in cardiac magnetic resonance imaging. Arch Cardiovasc Dis 2012; 105: 91–98. 22424327 10.1016/j.acvd.2012.01.002

[18] Alsaileek AA Osranek M Fatema K , et al. Predictive value of normal left atrial volume in stress echocardiography. JACC 2006; 47: 1024–1028. 16516088 10.1016/j.jacc.2005.09.069

[19] Choi JY Cha J Jung JM , et al. Left ventricular wall motion abnormality is associated with cryptogenic stroke. Int J Stroke 2020; 15: 188–196. 30982433 10.1177/1747493019834181

[20] Kim Y Kim TJ Lee SH . Cardiac wall motion abnormality as a predictor for undetermined stroke with embolic lesion-pattern. Clin Neurol Neurosur 2020; 191: 105677. 10.1016/j.clineuro.2020.105677 31958700

[21] Husic M Nørager B Egstrup K Møller JE . Usefulness of left ventricular diastolic wall motion abnormality as an early predictor of left ventricular dilation after a first acute myocardial infarction. Am J Cardiol 2005; 96: 1186–1189. 16253579 10.1016/j.amjcard.2005.06.053

[22] Thomas L Marwick TH Popescu BA , et al. Left atrial structure and function, and left ventricular diastolic dysfunction. JACC 2019; 73: 1961–1977. 31000000 10.1016/j.jacc.2019.01.059

[23] Kamran S Singh R Akhtar N , et al. Left heart factors in embolic stroke of undetermined source in a multiethnic Asian and North African Cohort. JAHA 2020; 9: e016534. 32750304 10.1161/JAHA.120.016534 PMC7792276

[24] Perera KS Vanassche T Bosch J , et al. Embolic strokes of undetermined source: prevalence and patient features in the ESUS Global Registry. Int J Stroke 2016; 11: 526–533. 27256472 10.1177/1747493016641967

[25] Galasko GIW Basu S Lahiri A Senior R . A prospective comparison of echocardiographic wall motion score index and radionuclide ejection fraction in predicting outcome following acute myocardial infarction. Heart 2001; 86: 271–276. 11514477 10.1136/heart.86.3.271 PMC1729882

[26] Carlhäll CJ Bolger A . Passing strange: flow in the failing ventricle. Circ Heart Fail 2010; 3: 326–331. 20233994 10.1161/CIRCHEARTFAILURE.109.911867

[27] Svalbring E Fredriksson A Eriksson J , et al. altered diastolic flow patterns and kinetic energy in subtle left ventricular remodeling and dysfunction detected by 4D flow MRI. PLoS One 2016; 11: e0161391. 27532640 10.1371/journal.pone.0161391 PMC4988651

[28] Gimbrone MA García-Cardeña G . Vascular endothelium, hemodynamics, and the pathobiology of atherosclerosis. Cardiovasc Pathol 2013; 22: 9–15. 22818581 10.1016/j.carpath.2012.06.006 PMC4564111

[29] Malek AM Alper SL Izumo S . Hemodynamic shear stress and its role in atherosclerosis. JAMA 1999; 282: 2035–2042. 10591386 10.1001/jama.282.21.2035

[30] Kumar S Williams D Sur S , et al. Role of flow-sensitive microRNAs and long noncoding RNAs in vascular dysfunction and atherosclerosis. Vascul Pharmacol 2019; 114: 76–92. 30300747 10.1016/j.vph.2018.10.001 PMC6905428

[31] Warboys CM Amini N de Luca A Evans PC . The role of blood flow in determining the sites of atherosclerotic plaques. F1000 Med Rep 2011; 3: 5. 21654925 10.3410/M3-5 PMC3096883

[32] Kojima K Kimura S Hayasaka K , et al. Aortic plaque distribution, and association between aortic plaque and atherosclerotic risk factors: an aortic angioscopy study. J Atheroscler Thromb 2019; 26: 997–1006. 30918164 10.5551/jat.48181 PMC6845689

[33] Hart RG Sharma M Mundl H , et al. Rivaroxaban for stroke prevention after embolic stroke of undetermined source. N Engl J Med 2018; 378: 2191–2201. 29766772 10.1056/NEJMoa1802686

[34] Hathcock JJ . Flow effects on coagulation and thrombosis. Arterioscler Thromb Vasc Biol 2006; 26: 1729–1737. 16741150 10.1161/01.ATV.0000229658.76797.30

[35] Garg P van der Geest RJ Swoboda PP , et al. Left ventricular thrombus formation in myocardial infarction is associated with altered left ventricular blood flow energetics. Eur Heart J Cardiovasc Imaging 2019; 20: 108–117. 30137274 10.1093/ehjci/jey121 PMC6302263

[36] Asinger RW Mikell FL Sharma B Hodges M . Observations on detecting left ventricular thrombus with two-dimensional echocardiography: emphasis on avoidance of false positive diagnoses. Am J Cardiol 1981; 47: 145–56. 7457401 10.1016/0002-9149(81)90303-9

[37] Takasugi J Yamagami H Noguchi T , et al. Detection of left ventricular thrombus by cardiac magnetic resonance in embolic stroke of undetermined source. Stroke 2017; 48: 2434–2440. 28818863 10.1161/STROKEAHA.117.018263

[38] Pritchett AM Mahoney DW Jacobsen SJ , et al. Diastolic dysfunction and left atrial volume: a population-based study. JACC 2005; 45: 87–92. 15629380 10.1016/j.jacc.2004.09.054

[39] Morris DA Belyavskiy E Aravind-Kumar R , et al. Potential usefulness and clinical relevance of adding left atrial strain to left atrial volume index in the detection of left ventricular diastolic dysfunction. JACC Cardiovasc Imaging 2018; 11: 1405–1415. 29153567 10.1016/j.jcmg.2017.07.029

[40] Hur J Kim YJ Lee HJ , et al. Left atrial appendage thrombi in stroke patients: detection with two-phase cardiac CT angiography versus transesophageal echocardiography. Radiology 2009; 251: 683–690. 19366905 10.1148/radiol.2513090794

[41] Meisel K Yuan K Fang Q , et al. Embolic stroke of undetermined source: a population with left atrial dysfunction. J Stroke Cerebrovasc Dis 2019; 28: 1891–1896. 31031144 10.1016/j.jstrokecerebrovasdis.2019.04.004

[42] Kamel H Longstreth WT Tirschwell DL , et al. The atrial cardiopathy and antithrombotic drugs in prevention after cryptogenic stroke randomized trial: rationale and methods. Int J Stroke 2019; 14: 207–214. 30196789 10.1177/1747493018799981 PMC6645380

[43] Geisler T Poli S Meisner C , et al. Apixaban for treatment of embolic stroke of undetermined source (ATTICUS randomized trial): rationale and study design. Int J Stroke 2017; 12: 985–990. 27881833 10.1177/1747493016681019

[44] Diener H-C Sacco RL Easton JD , et al. Dabigatran for prevention of stroke after embolic stroke of undetermined source. N Engl J Med 2019; 380: 1906–1917. 31091372 10.1056/NEJMoa1813959

[45] Eikelboom JW Connolly SJ Bosch J , et al. Rivaroxaban with or without aspirin in stable cardiovascular disease. N Engl J Med 2017; 377: 1319–1330. 28844192 10.1056/NEJMoa1709118

