# Supplementary material for: Partitioning risk factors for embolic stroke of undetermined source using exploratory factor analysis
Source: Int J Stroke. 2021 Apr 26;17(4):407–14. doi: 10.1177/17474930211009847 (PMC8969073; doi:10.1177/17474930211009847)
Supplement: sj-pdf-1-wso-10.1177_17474930211009847 - Supplemental material for Partitioning risk factors for embolic stroke of undetermined source using exploratory factor analysis [file sj-pdf-1-wso-10.1177_17474930211009847.pdf]

SUPPLEMENTARY MATERIAL A

Further statistical methods

Exploratory factor analysis (EFA) was applied to each data set using M*plus* version 6.12 which used pairwise deletion for missing data.[1] EFA was used to assess the relationships between observed variables and to investigate unobserved latent factors. As data was categorical, polychoric correlation matrices were estimated using mean- and variance-corrected weighted least squares (WLSMV). WLSMV does not rely on the model covariance matrix being positive definite and is therefore a robust way to explore non-normally distributed data.[2] To further clarify the relationship between variables an oblique rotation was performed using Geomin method. Oblique method was used as it was assumed latent variables would correlate. The number of factors to retain was determined using Kaiser-Guttmann criterion (eigenvalues ≥1) in addition to visual inspection of the scree test.[3] Fit statistics with cut-off values were also used, such as, Comparative Fit Index (CFI, ≥.90 acceptable, ≥.95 excellent), Tucker Lewis Index (TFI, ≥.90 acceptable, ≥.95 excellent), and root mean square error of approximation (RMSEA, ≤.08 acceptable, ≤.05 excellent).[4,5] A latent factor was considered acceptable if it included a minimum of three or more indicator variables.[6] Inclusion was based on indicators having a weighting of ≥.3 as guided by sample size.[8] Where cross loading occurred (defined as weighting of ≥.3 on more than one latent factor), items were removed sequentially and the analysis repeated until a satisfactory solution was found.[9] To understand the contribution of latent factors to the model the percentage of eigenvalues is presented. This is because WLSMV is based on correlation rather than variance and M*plus* does not provide these data.[1] After identification of relevant indicators, the analyses were rerun using WLSMV

specifying model parameters so that factor determinacy could be calculated. Factor determinacy reflects the correlation between estimated and true factor scores and ranges between 0-1. In addition, correlation between latent variables was generated.

Supplementary Results

In the ESUS group, Kaiser-Guttman criterion indicated six factors should be retained with eigenvalues of 2.88, 1.70, 1.36, 1.18 1.12 and 1.04. The first three values accounted for 22.2 %, 13.1% and 10.5% of the sum of eigenvalues and 45.8% overall. However, it has been suggested that eigenvalues overestimate the number of factors[28] and the scree plot hinted at 2 or 3 factors. The first ESUS analysis was run with all indicator variables added to the model and 1-, 2-, and 3-factor solutions considered. Fit statistics were excellent for all factor solutions (Supplementary Table 1). However, the 3-factor solution was rejected as one latent factor loaded with only two indicator variables suggesting over-factoring. The next best solution was for 2-factors but ‘smoking’ cross-loaded on both latent variables (0.36 and 0.39) and was therefore removed. After repeating the analysis, a satisfactory 2-factor model was confirmed for ESUS (χ^2=56.4, df, 53, RMSEA, 0.01, CFI, 1.00, TFI, 1.00). The first latent factor, “Cardiac 1”, consisted solely of heart related features. There were three contributing indicators and the strongest loading was LVWMA (-0.98), which was negatively correlated specifying that focal and global LVWMA were important contributors (LVWMA were coded 0-2 for none, focal and global). The next highest contributor to Cardiac 1 was EF (0.71) followed by LAVI (0.48). The second latent factor, “Cardiac 2”, was also dominated by heart related attributes. There were four contributing indicators, dyslipidaemia (-0.43), aortic arch atherosclerosis (AAA) (0.50), LVDD (0.36), LVMI (0.32). Dyslipidaemia loaded negatively suggesting abnormal blood

lipids, are not involved in ESUS pathology. AAA was the dominant contributor to Cardiac 2 and was the only positively associated non-heart feature found in ESUS patients. The correlation between Cardiac 1 and Cardiac 2 on the specified model was a moderate 0.34 and factor determinacies ranged from very good to good at 0.98 for Cardiac 1 and 0.74 for Cardiac 2 (Supplementary Table 2).

In the all other strokes data, Kaiser-Guttman criterion indicated seven factors should be retained with eigenvalues of 2.59, 1.73, 1.57, 1.33, 1.13, 1.07, and 1.02. The first three eigenvalues accounted for 19.9%, 13.3% and 12.1% of the sum of the eigenvalues and 45.3% overall. Visual analysis of the scree plot indicated either five or possibly seven factors should be retained. As seven factors was likely an overestimation, 1-5-factor solutions were explored in the first all other strokes analysis with all indicator variables included. For 4- and 5- factor solutions, iterations were exceeded, and the models failed to converge. Fit statistics ranged from good to excellent for solutions 1-3 (Supplementary Table 1). The 3-factor model was excluded as only two indicator variables loaded on the third latent factor. For the next best fit (2-factors), two indictors cross loaded, mitral calcification (MITCAL) and smoking, and were deleted sequentially. Following the removal of MITCAL, smoking resolved and was included in the accepted 2-factor model (χ^2=64.7, df, 53, RMSEA, 0.03, CFI, 0.98, TFI, 0.97). The first factor “AOS Heart” mirrored the ESUS data and included LVWMA (1.0) followed by EF (-0.83) and LAVI (0.48). Both LVWMA and EF loaded in the opposite direction compared to the ESUS data meaning that normal or focal/normal or mild, LVWMA/EF, clustered with LAVI in these patients. A second latent factor in all other strokes “Traditional” consisted entirely of tradition risk factors. The top contributor was smoking (0.50), followed by HTN (0.48), dyslipidaemia (0.43) and DM (0.32). The correlation between AOS Heart and

Traditional factors on the specified model was small at 0.12 and factor determinacies were strong for both at 0.97 and 0.73 respectively (Supplementary Table 2).

Supplementary table 1. Fit statistics for ESUS and all other strokes exploratory factor analyses

Latent variables	Fit statistic				
**ESUS Group**	χ^2	*df*	**RMSEA**	**CFI**	**TLI**
1-factor model	102.5	77	0.03	0.98	0.98
2-factor model	67.3	64	0.01	1.00	1.00
**2-factor model***	**56.4**	**53**	**0.01**	**1.00**	**1.00**
3-factor model	50.0	52	0.01	1.00	1.00
**AOS Group**					
1-factor model	109.5	77	0.04	0.94	0.93
2-Factor model	84.9	64	0.03	0.96	0.95
**2-factor model[§]**	**64.7**	**53**	**0.03**	**0.98**	**0.97**
3-Factor model	65.0	52	0.03	0.98	0.96
4-Factor model	Non-convergence				
5-Factor model	Non-convergence				

Note. N =331. ESUS= embolic stroke of undetermined source, AOS=all other strokes, χ^2=model Chi-square, *df*=degrees of freedom, RMSEA=root mean square error of approximation, CFI = comparative fit index, TLI = Tucker Lewis Index.

[*]Final model without Smoking.

[§]Final model without Mitral calcification.

Supplementary Table 2. Eigenvalues, factor determinacies and correlations for ESUS and all other stroke groups

	**ESUS**		**AOS**	
	**Cardiac 1**	**Cardiac 2**	**AOS Heart**	**Traditional**
**Eigenvalue**	2.73	1.56	2.36	1.73
**FD**	.98	.74	.97	.73
**r-Cardiac 1**	1.0	.34	-	-
**r-AOS Heart**	-	-	1.0	.12

Note. ESUS= embolic stroke of undetermined source, AOS=all other strokes, FD= factor determinacies. r=correlation. Both FD and r are derived from the specified WLSMV model.

References

1. Muthén, LK., & Muthén, BO. (1998-2011). *Mplus User's Guide*. Sixth Edition. Los Angeles, CA: Muthén & Muthén.

2. Muthén, BO., du Toit, SHC., & Spisic, D. (1997). Robust inference using weighted least squares and quadratic estimating equations in latent variable modeling with categorical and continuous outcomes. Retrieved from http://gseis.ucla.edu/faculty/muthen/articles/Article_075.pdf

3. Cattell, RB. (1966). The Scree Test For The Number Of Factors. *Multivariate Behavioral Research*, *1*(2), 245–276.

4. Bentler, PM. (1990). Comparative fit indexes in structural models. *Psychological Bulletin*, *107*(2), 238–246.

5. Browne, MW, & Cudeck, R. (1993). Alternative Ways of Assessing Model Fit. In K. A. Bollen, & JS. Long (Eds.), *Testing Structural Equation Models* (pp. 136-192). Newbury Park, CA: Sage.

6. Osborne, JW., & Costello, AB. (2005). Best Practices in Exploratory Factor Analysis: Four Recommendations for Getting the Most From Your Analysis. *Practical Assessment, Research & Evaluation,* 1–9.

8. Hair, JF., Black WC., Babin BJ., and Anderson RE. (2010), Multivariate Data Analysis, Englewood, Cliffs, NJ: Prentice Hall.

9. Tabachnick, BG., & Fidell, LS. (2001), *Using Multivariate Statistics* (5th ed.). Pearson: Needham Heights, MA.
